# Exploring infant gut–derived probiotics through 16S rRNA identification and in vitro functional characterization

**DOI:** 10.1371/journal.pone.0343741

**Published:** 2026-02-25

**Authors:** Ishita Modasiya, Ritesh Nasit, Harsh Purohit, Apurba Kumar Sarkar, Vijay Kumar

**Affiliations:** 1 Postbiotics and Foodomics Lab, Department of Microbiology, School of Science, RK University, Rajkot, Gujarat, India; 2 Aegis Lifesciences Pvt Ltd, Ahmedabad, Gujarat, India; 3 Paradise Scientific Company Ltd, Dhaka, Bangladesh; 4 Department of Microbiology and MLT, Bhagwan Mahaveer College of Applied Science, Bhagwan Mahaveer University, Surat, Gujarat, India; Indian Institute of Technology Madras, INDIA

## Abstract

The infant gut microbiota significantly influences health outcomes, impacting digestion, immunity, and metabolic processes. Identifying beneficial microbes from this source offers promising avenues for novel probiotic development. This study aimed to identify and characterize potential probiotic bacterial isolates from infant fecal samples using 16S rRNA–based identification and in vitro experimental evaluations. A total of 47 isolates were initially screened. Two promising candidates, *Lb. paracasei* IF5YR and *Ent. faecium* IF5RW2, were selected and evaluated for gastrointestinal resilience (acid and bile tolerance), functional properties (autoaggregation, cell surface hydrophobicity, bile salt hydrolase activity, antioxidant activity via FRAP, ABTS, and DPPH assays), and safety (antibiotic susceptibility). Their genetic affiliations were confirmed using 16S rRNA sequencing, followed by bioinformatics and phylogenetic analyses. Both *Lb. paracasei* IF5YR and *Ent. faecium* IF5RW2 demonstrated strong acid (pH 1.5) and bile (2%) tolerance, high autoaggregation (>70%), and notable cell surface hydrophobicity (>90% for xylene). Comparative analysis of antibiotic susceptibility confirmed their safety. Phylogenetic analysis based on 16S rRNA sequencing supported the taxonomic affiliation of the isolates. Functionally, both strains exhibited bile salt hydrolase activity and strong antioxidant potential, with FRAP values exceeding 75%. These findings suggest *Lb. paracasei* IF5YR and *Ent. faecium* IF5RW2 may be considered potential candidates for probiotic applications related to gut health and oxidative stress modulation. They hold potential for development as novel functional food ingredients or health-promoting supplements, although *in vivo* or clinical studies are needed to confirm any therapeutic effects.

## 1 Introduction

The intricate ecosystem of the human gastrointestinal tract, teeming with trillions of microorganisms, plays an indispensable role in maintaining health, modulating immunity, and defending against pathogens [1]. The establishment of this microbial community during early life is particularly critical, as neonatal colonization patterns are strongly shaped by delivery mode and infant diet. For example, vaginally delivered infants typically acquire beneficial *Lactobacillus* and *Bifidobacterium* strains, while Cesarean-delivered infants often exhibit reduced microbial diversity, predisposing them to long-term health risks [2]. Early microbial colonization has been associated with long-term health outcomes, including susceptibility to inflammatory, metabolic, and oxidative stress–related disorders [3,4].

Amid growing scientific and consumer interest, probiotics have emerged as a cornerstone for modulating gut microbial balance and promoting host health. Probiotics, defined as “live microorganisms that, when administered in adequate amounts, confer a health benefit on the host,” have been shown to enhance immune defences, antagonize pathogenic bacteria, reduce cholesterol, and counteract oxidative stress [5]. Dairy matrices remain among the most effective and acceptable carriers of probiotic strains, supporting their survival and broad consumer acceptance. Of special interest are human-origin probiotics, which may exhibit improved colonization potential and functional compatibility within the human gastrointestinal tract compared to strains sourced from plants or animals [2]. Their application extends to innovative therapeutic strategies like faecal microbiota transplantation (FMT) for gut dysbiosis [6], with their antioxidant capabilities, derived from enzymatic systems and bioactive compounds like exopolysaccharides, being vital for neutralizing free radicals and safeguarding cellular integrity [7,8].

While a wide array of *Lacticaseibacillus paracasei* and *Enterococcus faecium* strains has already been reported in the literature, most are sourced from fermented foods or adult-derived microbiota. Infant-origin isolates remain comparatively underexplored despite their adaptation to the early-life gut environment – a unique niche associated with robust colonization potential and distinct functional traits. This study, therefore, extends beyond merely reaffirming regional probiotic diversity. By integrating comparative genomic analyses with functional assays, we address two key knowledge gaps: (i) identifying infant-derived strains with probiotic potential that differ physiologically from food- or adult-derived isolates, and (ii) characterizing a constellation of traits – remarkable gastrointestinal tolerance (acid and bile), strong adhesive properties (autoaggregation, hydrophobicity), enzymatic bile salt hydrolase activity, and superior antioxidant potential, that have not been comprehensively reported together for *L. paracasei* or *E. faecium*.

Accordingly, from 47 infant fecal isolates, we identified and characterized two promising candidates: *L. paracasei* IF5YR and *E. faecium* IF5RW2. The isolates were identified by 16S rRNA–based phylogenetic analysis and experimentally evaluated for functional, safety, and antioxidant properties. By demonstrating the distinctive probiotic and antioxidant attributes of these infant gut-derived strains, this study positions them as novel candidates for dairy-based functional foods and therapeutic probiotic formulations with targeted roles in gut health and oxidative stress management.

## 2 Materials and methods

### 2.1 Ethics statement, isolation and characterization, and safety assessment of infant faeces

The RK University School of Physiotherapy Ethics Committee approved this study (Ref. No.: RKU/SPT/2021/07/07). Participants (children) were prospectively recruited for the study between 20/07/2021 and 14/04/2022. During this period, isolation of bacterial strains from faecal samples was also completed. Written informed consent was obtained from the parents or legal guardians of all minor participants (children) before sample collection. The consent form explained the study’s purpose, procedures, potential risks, and confidentiality protections. All participants’ guardians signed the consent form. Verbal assent from children was not applicable due to their age group. No waiver of consent was obtained or requested.

Ten healthy infants aged 2–24 months (8 boys, 2 girls), 5 were born by caesarean delivery (C-section) and 5 by vaginal delivery. Fecal samples (∼1 g) were collected directly from diapers or the infant’s skin using a sterile scoop, into sterile containers, and stored at −20°C in the RK University, Biotechnology Lab (Department of Microbiology), Rajkot, Gujarat, until further analysis. Faecal samples from infants were handled and processed individually. For isolation, samples were diluted with peptone water and spread onto de Man-Rogosa-Sharpe (MRS) agar plates. Plates were incubated under optimal conditions at 37°C for 24 h in an anaerobic environment. The bacterial isolates obtained were initially screened based on colony morphology, microscopic characteristics, and basic biochemical tests, including oxidase and catalase tests.

### 2.2 Antibiotic susceptibility test

The susceptibility of bacterial isolates to five clinically relevant antibiotics (ciprofloxacin, erythromycin, penicillin, tetracycline, and vancomycin) was assessed through a standardized disk diffusion method. Isolates grown on MRS agar plates with antibiotic-infused disks form clear zones after incubation at 37°C for 24 h [9]. The size of clear zones around the disk, as per the Clinical Laboratory Standards Institute (CLSI) guidelines (2017), [10] indicated the susceptibility or resistance of isolates to the specific antibiotic. This evaluation is crucial for ensuring the safety of potential probiotic candidates.

### 2.3 Comparative analysis of antibiotic susceptibility patterns

A heatmap was generated to compare antibiotic susceptibility patterns across various bacterial species, including strains from the current study (*Lb. paracasei IF5YR* and *Ent. faecium IF5RW2*) and reference strains from previous research, including *S. thermophilus HN2* and *HN3* (Nirvan et al., 2024), *L. casei*, *L. paracasei*, *L. pentosus*, and *L. delbrueckii* (Kumar et al., 2022), *P. pentosaceus DM101* (Rappai et al., 2020), and *E. durans LAB18s* (Archer et al., 2019). Five antibiotics were tested: Ampicillin (AMP), Ciprofloxacin (CIP), Erythromycin (ERY), Tetracycline (TET), and Streptomycin (STR). The heatmap shows the Zone of Inhibition (ZOI) in millimeters (mm) without rounding, providing precise comparison across species. A color gradient from light yellow to dark green was used, with darker shades indicating higher sensitivity. This comparative analysis provided the antibiotic susceptibility profiles of the tested strains, emphasizing the potential sensitivity of *Lb. paracasei IF5YR* and *Ent. faecium IF5RW2* to CIP and ERY compared to other species.

### 2.4 Haemolytic activity

Activated bacterial isolates were streaked onto blood agar plates containing 5% sheep blood. The plates were then incubated for 24 h at 37°C. After incubation, clear zone surrounding the isolates indicated β-haemolytic positive, a greenish zone indicated α-haemolysis, and the absence of a zone indicates no haemolysis or γ-haemolysis.

### 2.5 Phylogenetic analysis of study sequences

A phylogenetic tree was constructed using the Maximum Likelihood (ML) method in MEGA11 to investigate the evolutionary relationships of 16S rRNA sequences obtained from the study isolates with known probiotic bacteria. The analysis employed the Tamura-Nei substitution model to account for transition/transversion rate bias and nucleotide frequency differences. Uniform rates were assumed across all sites, and all nucleotide positions in the 16S rRNA sequences included in the phylogenetic analysis to maximize phylogenetic information. The tree topology was optimized using the Nearest-Neighbor-Interchange (NNI) heuristic method, and the reliability of the branches was assessed with 100 bootstrap replicates. The resulting phylogenetic tree illustrates the evolutionary placement of the study isolates in relation to established probiotic strains, providing insights into their potential phylogenetic relatedness.

### 2.6 Comparative analysis of acid tolerance and bile tolerance

Studying the ability of isolates to survive acidic conditions and high bile concentration in the stomach is crucial for selecting probiotic cultures. Both attributes were performed following the novel spot plate method mentioned in the Modasiya et al. [11] to assess the viability of isolates at various pH levels (1.5, 2, 3, 4, and 6.8) and bile concentrations (0.5%, 1%, 1.5%, 2%).

To compare acid and bile salt tolerance across different bacterial species, survivability was measured at three pH levels (1.5, 2.0, and 3.0) and four bile concentrations (0.3%, 0.6%, 1.0%, and 2.0%). The strains tested included *Lb. paracasei* IF5YR and *Ent. faecium* IF5RW2 from the current study, as well as individual reference strains from previous research: *S. thermophilus* HN2 and HN3 [12], *L. fermentum* N7, *L. plantarum* N17, and *L. paracasei* N27 [13], *P. pentosaceus* F35-3 and D1 [14], *E. durans* LAB18s [15], *L. fermentum* IF199 (Infant 4 Study), and *L. plantarum* P1, *L. fermentum* P10, P193 [16]. Survivability was calculated as the percentage of viable cells remaining after exposure to each condition. Italicized names were used for scientific accuracy, and text wrapping was applied for better readability. To ensure consistency and fairness in comparison, only one top-performing strain from each reference study, specifically the one that exhibited the highest survivability at the most extreme pH and bile conditions tested, was included in the graph.

### 2.7 Cell autoaggregation and cell surface hydrophobicity

The autoaggregation potential of isolates was evaluated using the protocol of [17]. After washing and resuspending the cells in phosphate buffer solution (PBS), the optical density (O.D.) at 600 nm was monitored for 5h. A decrease in O.D. indicated aggregation as the cell suspension clears. Autoaggregation (%) was calculated as, A% = (1 - OD_final_/ OD_initial_) × 100, where A = autoaggregation, OD_initial_ = cell suspension O.D. before incubation, OD_final_ = cell suspension O.D. at 5^th^ h. Autoaggregation was calculated as the percentage of self-aggregation, indicating the strain’s potential to form stable biofilms.

The microbial adhesion to hydrocarbons (MATH) method [18] was employed to quantify the hydrophobicity of the isolated cultures. This method assesses the tendency of cultures to stick to hydrocarbons, indicating their affinity for non-polar environments. Higher adhesion signifies greater hydrophobicity. The experiment measured the O.D. at 610 nm before and after hydrocarbon exposure (xylene, toluene). Hydrophobicity was measured as the percentage of adhesion to hydrocarbons, reflecting the strain’s ability to adhere to intestinal epithelial cells. The percent hydrophobicity (H%) was then calculated using the formula: H% = (OD_initial_ – OD_final_)/ OD_initial_ × 100.

### 2.8 Comparative analysis of hydrophobicity and autoaggregation

Hydrophobicity and autoaggregation were compared across *Lb. paracasei* IF5YR and *Ent. faecium* IF5RW2 from the current study, along with reference strains from previous research: *S. thermophilus* HN2 and HN3, *Lb. casei*, *Lb. paracasei*, *Lb. pentosus*, *Lb. delbrueckii*, *P. pentosaceus* DM101, and *Ent. durans* LAB18s. A graph was created to visualize the percentages. Italicized bacterial names were used for scientific accuracy, and longer strain names were wrapped for better readability.

### 2.9 Bile salt hydrolase activity

A qualitative plate assay was used to evaluate the ability of probiotic strains to deconjugate bile salts [19]. The assay involved preparing modified MRS media by adding 0.5% (w/v) bile salts (sodium deoxycholate and sodium taurocholate) and 0.037% calcium chloride (CaCl_2_). Normal MRS plates served as the control for the experiments. A clear zone or opaque halo surrounding colonies on the modified plates indicated bile salt deconjugation by the probiotic cultures.

### 2.10 Antioxidant activity

The antioxidant potential of the isolates was evaluated using cell-free extracts (CFE) [20]. Overnight-grown cultures were inoculated into fresh MRS broth and incubated at 37°C for 24 h. Following centrifugation at 10,000 rpm for 5 minutes at 4°C, the supernatant was collected and filtered through a 0.22 µm syringe filter. These extracts were then subjected to Azino-Bis (3-Ethyl-Benzothiazoline) 6-Sulfonic Acid (ABTS) [21], 2, 2-Diphenyl-1-Picrylhydrazyl (DPPH) [22], and Ferric-Reducing Antioxidant Potential (FRAP) [23] assays to assess their free radical scavenging abilities.

ABTS (7 mM) and potassium persulfate (2.45 mM) were combined and incubated for 14–16 hours in the dark to generate ABTS radicals. The solution was diluted with PBS to an OD of 0.70 ± 0.02 at 734 nm (Shimadzu-UV1900). A 990 μL aliquot of the ABTS solution was mixed with 10 μL of CFE, and absorbance changes at 734 nm were recorded at 30-second intervals over 6 minutes. Radical scavenging activity was calculated as [(A_734_ initial − A_734_ final)/ A_734_ initial] × 100. Ascorbic acid (100–1000 μM) served as the standard for the assay.

A 5 mM DPPH solution was prepared in 70% methanol, adjusted to an OD of 0.70 ± 0.02 at 515 nm (Shimadzu-UV1900). For the assay, 20 μL of CFE was added to 980 μL of the DPPH solution. Absorbance was measured at 515 nm at 30-second intervals for 6 minutes. Radical scavenging activity was calculated as [(A_515_ initial − A_515_ final)/ A_515_ initial] × 100. Ascorbic acid (100–1000 μM) was used to generate the standard curve.

The FRAP reagent was freshly prepared by mixing 300 mM acetate buffer, 8 mM TPTZ dissolved in 40 mM HCl, and 20 mM FeCl₃ in a 10:1:1 ratio. For the assay, 50 μL of cell-free extract (CFE) was combined with 1.5 mL of FRAP reagent and ultrapure water to reach a total volume of 1.7 mL. After incubation at 37°C for 10 minutes in the dark, the absorbance was measured at 593 nm using a UV spectrophotometer (Shimadzu-UV1900). Ferrous sulphate (25–400 μM) was used to generate the standard curve, and reducing power was calculated accordingly.

### 2.11 Statistical analysis

Statistical comparisons of various quantitative data obtained were carried out using one-way and two-way analysis of variance (ANOVA) by Tukey test with the help of GraphPad Prism version 8. The differences between the means were compared using a one-way and two-way ANOVA with a significance level of P < 0.05. All assays were performed in three independent experiments and results are expressed as Mean± Standard deviation (SD), taking n equals to three.

## 3 Results

### 3.1 Isolation, characterization, and safety assessment of LAB from infant feces

Following ethical guidelines, stool samples were obtained from 10 healthy infants aged 2–24 months, irrespective of delivery mode. A total of 47 microbial isolates were obtained from these samples, which were subjected to primary screening. These isolates were identified as Gram-positive, rod- and cocci-shaped, catalase-negative, and oxidase-negative, with sizes ranging between 0.3 to 0.9 μM. These isolates were later investigated for antibiotic susceptibility and haemolytic ability to ensure their safety.

The antibiotic susceptibility of these isolates was evaluated according to CLSI guidelines. Among the 47 isolates, 13 exhibited complete resistance to all five tested antibiotics, while five showed resistance to three antibiotics. Ten isolates displayed intermediate sensitivity to vancomycin but were resistant to the remaining four antibiotics. Seven isolates (IF4R2, IF5YR, IF5RW2, IF5SR2, IF5SR3, IF7RW1, IF7IR2) that showed sensitive or intermediate sensitive response to more than two antibiotics were selected for further studies.

Among the seven selected isolates, two isolates (IF5SR2, IF7IR2) showed β haemolysis, characterized by a complete clearing zone around the bacterial growth on a blood agar plate, indicating a stronger haemolytic potential. The remaining five isolates (IF4R2, IF5YR, IF5RW2, IF5SR3, IF7RW1), that displayed non-haemolytic activity, were selected for further investigation to determine their suitability for probiotic applications.

### 3.2 Phylogenetic analysis of 16S rRNA sequences

**Fig 1:**
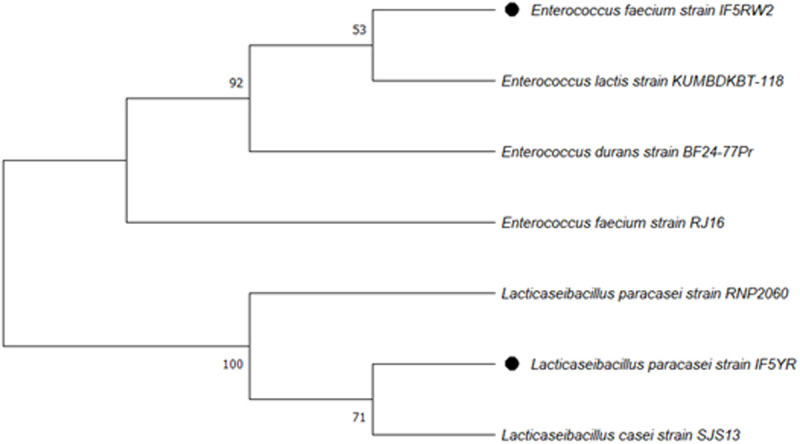
Phylogenetic tree of 16s rRNA sequences showing evolutionary relationships of study isolates with probiotic bacteria.

The phylogenetic tree (Fig 1) shows that the study isolates are grouped into two main clades: *Enterococcus* and *Lacticaseibacillus*. The *Enterococcus* clade includes *Ent. faecium* (IF5RW2, RJ16), *Ent. lactis* (KUMBDKBT-118), and *Ent. durans* (BF24–77Pr), supported by high bootstrap values (92% and 53%), indicating a close evolutionary relationship within this genus. The *Lacticaseibacillus* clade groups *Lb. paracasei* (RNP2060, IF5YR) and *Lb. casei* (SJS13) with a strong bootstrap value (100%), confirming high evolutionary conservation. These findings suggest that the study isolates are phylogenetically related to known probiotic strains, highlighting their potential as probiotic candidates. Details of the isolate names and their GenBank accession numbers are provided in Table 1, ensuring traceability and accessibility of the genetic data for future studies.

**Table 1:** Candidate probiotics were identified based on the 16s rRNA sequence to those available in the GenBank database.

Isolates code	Identified as	Accession No.
IF5YR	*Lb. paracasei* IF5YR	PP237494
IF5RW2	*Ent. faecium* IF5RW2	PP237491

### 3.3 Comparative analysis of antibiotic susceptibility

**Fig 2:**
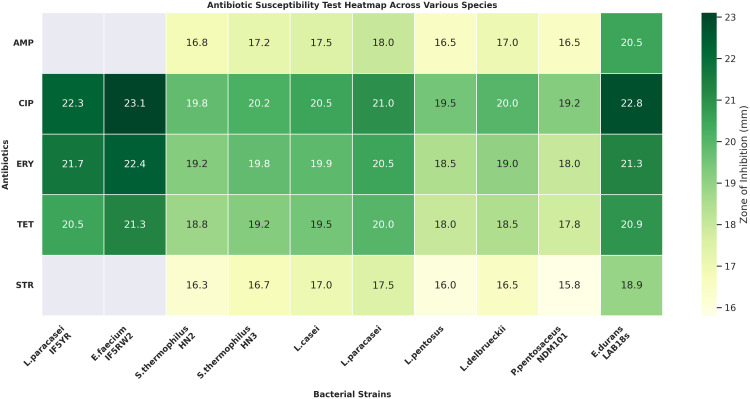
Antibiotic susceptibility test heatmap across various species. The heatmap displays the Zone of Inhibition (ZOI) values (in mm) for six antibiotics: Ampicillin (AMP), Ciprofloxacin (CIP), Erythromycin (ERY), Tetracycline (TET), and Streptomycin (STR). Darker shades of green indicate higher sensitivity. Italicized names represent the tested bacterial strains, including those from the current study (*Lb. paracasei* IF5YR and *Ent. faecium* IF5RW2) and reference strains from previous studies.

The heatmap compares antibiotic susceptibility across bacterial species, including *Lb. paracasei* IF5YR and *Ent. faecium* IF5RW2 from the current study and reference strains from previous research. Six antibiotics were tested: Ampicillin (AMP), Ciprofloxacin (CIP), Chloramphenicol (CHL), Erythromycin (ERY), Tetracycline (TET), and Streptomycin (STR) (Fig 2). The results showed that *Lb. paracasei* IF5YR and *Ent. faecium* IF5RW2 were highly sensitive to ciprofloxacin and erythromycin, and moderately sensitive to tetracycline, indicating a favourable antibiotic susceptibility profile and supporting their biosafety as potential probiotic candidates. In contrast, *S. thermophilus* (*HN2* and *HN3*) showed lower sensitivity across all antibiotics, suggesting a higher resistance profile. Species-specific differences were noted within the genus *Lactobacillus*, where *L. pentosus* and *L. delbrueckii* showed lower sensitivity to TET and ERY compared to *Lb. paracasei*. Meanwhile, *P. pentosaceus DM101* and *E. durans* LAB18s demonstrated moderate to high sensitivity to CIP and ERY, but lower sensitivity to AMP. Overall, the comparative analysis provides the antibacterial potential of *Lb. paracasei* IF5YR and *Ent. faecium* IF5RW2, particularly against CIP and ERY, supporting their use as effective probiotics against antibiotic-resistant pathogens.

### 3.4 Gastrointestinal survival

After a comprehensive safety evaluation, five isolates were selected for acid tolerance testing. Their resilience under highly acidic (pH 1.5) and bile-rich conditions were assessed using the spot plate method. Among the five isolates, IF5YR and IF5RW2 exhibited exceptional survival and growth at pH 1.5 after 4 hours of incubation (Table 2). These results indicated notable acid tolerance, leading to further evaluation of bile tolerance across five concentrations.

**Table 2:** *In vitro* acid tolerance and bile tolerance of isolates.

Cultures	Time (h)	Growth at different pH					Growth at different bile concentrations (w/v %)				
		6.8	4.0	3.0	2.0	1.5	0	0.5	1.0	1.5	2.0
***Lb. paracasei*** IF5YR	0	+++	++	++	++	++	++	++	++	++	++
	4	+++	++	++	++	–	+++	+++	+++	+++	+++
***Ent. faecium*** IF5RW2	0	+++	++	++	+	–	++	++	++	++	++
	4	+++	++	++	+	–	+++	+++	+++	+++	+++

Note: The data presented here is qualitative and is based on the results of experiments performed in triplicates. Number of “+” represents the noticeable growth as visible to moderate visible to transparent and “-” represents no growth of isolate on MRS agar.

Both IF5YR and IF5RW2 displayed consistent growth under simulated gastrointestinal conditions, maintaining their viability even at a high bile concentration of 2% after 4 hours (Table 2). Their ability to thrive in such challenging environments highlights their potential adaptability and survivability within the gastrointestinal tract. Comparative analysis against reference strains (*Lb. plantarum* NCDC 347 and *Lb. rhamnosus* NDRI 184) demonstrated that IF5YR and IF5RW2 exhibited superior tolerance, further establishing their resilience and suitability as probiotics [11].

### 3.5 Result of comparative analysis of acid and bile salt tolerance

**Fig 3:**
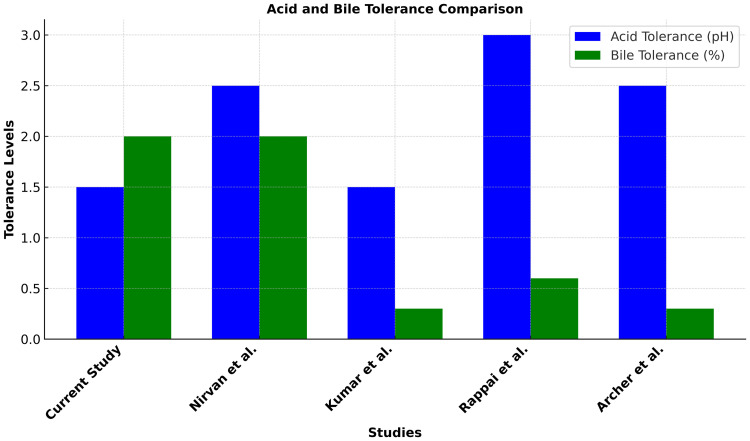
Comparative analysis of acid and bile salt tolerance across various species. The heatmap displays survivability percentages at pH levels 1.5, 2.0, and 3.0 and bile concentrations of 0.3%, 0.6%, 1.0%, and 2.0%. Italicized names represent the tested bacterial strains, including those from the current study (*Lb. paracasei* IF5YR and *Ent. faecium* IF5RW2) and one selected strain from each reference study based on maximum tolerance performance.

The comparative analysis revealed significant differences in acid and bile salt tolerance among the tested strains. *Lb. paracasei* IF5YR and *Ent. faecium* IF5RW2 demonstrated the highest survivability at pH 1.5 and 2.0 and maintained strong tolerance at 2.0% bile, indicating excellent gastrointestinal resilience. In contrast, *S. thermophilus* HN2 and HN3 showed limited tolerance under low pH and high bile conditions. Among other Lactobacillus strains, *L. pentosus* and *L. delbrueckii* showed lower survivability compared to *Lb. paracasei* and *Lb. fermentum*. The results confirm that *Lb. paracasei* IF5YR and *Ent. faecium* IF5RW2 outperform most reference strains in tolerating gastrointestinal-like conditions. Each strain included in the graph was tested individually, and the most acid- and bile-tolerant isolate from each study was selected for comparison (Fig 3).

### 3.6 Adhesion and colonization

The ability of probiotic strains to auto-aggregate is a critical attribute that reflects their potential to adhere to epithelial surfaces and establish colonization within the gastrointestinal tract. This property enables the formation of stable bacterial aggregates, enhancing their ability to outcompete pathogenic microorganisms. In a 5-hour assessment, both *Lb. paracasei* IF5YR and *Ent. faecium* IF5RW2 demonstrated strong autoaggregation capacities, surpassing the 70% benchmark considered optimal for probiotics.

**Fig 4:**
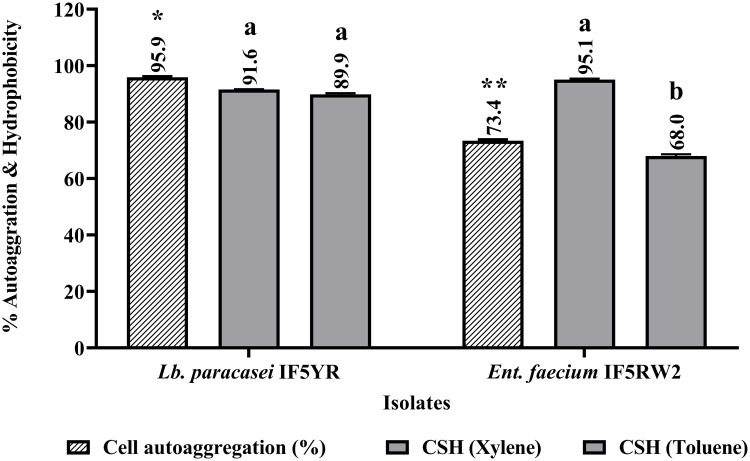
*In-vitro* cell autoaggregation and cell surface hydrophobicity of isolates. Data are expressed as % of auto-aggregation measured after 5 h of incubation. Values are presented as Mean±SD. abc*P < 0.05: significantly different. Bars with common superscripts represent non-significance in the property.

Notably, *Lb. paracasei* IF5YR exhibited an exceptional autoaggregation percentage of 95.9 ± 0.3%, significantly higher than *Ent. faecium* IF5RW2, which achieved 73.4 ± 0.4% (p < 0.05) (Fig 4). While the reference strains displayed slightly higher values [11], the observed results highlight the remarkable adherence potential of these isolates, particularly *Lb. paracasei* IF5YR. These findings underscore the suitability of both isolates for probiotic applications, emphasizing their ability to adhere to host epithelial surfaces and promote gut health by inhibiting pathogen colonization.

To further investigate their adhesion properties, cell surface hydrophobicity of *Lb. paracasei* IF5YR and *Ent. faecium* IF5RW2 was evaluated using xylene and toluene as representative hydrophobic solvents. Hydrophobicity is a critical determinant of microbial adherence to epithelial surfaces, directly influencing colonization and persistence within the gastrointestinal environment.

Both isolates exhibited a strong affinity for xylene, with hydrophobicity values of 91.6 ± 0.2% for *Lb. paracasei* IF5YR and 95.1 ± 0.4% for *Ent. faecium* IF5RW2. These high values highlight their robust hydrophobic interaction with xylene, underscoring their potential for effective epithelial binding. In contrast, the affinity for toluene was comparatively lower but still notable, with hydrophobicity values of 89.9 ± 0.4% for *Lb. paracasei* IF5YR and 68.0 ± 0.6% for *Ent. faecium* IF5RW2. A significant difference (p < 0.05) was observed between the two isolates in their interaction with toluene, indicating strain-specific variations in cell surface properties. However, no significant difference was detected in their interaction with xylene, suggesting a consistent hydrophobicity pattern toward this solvent. These findings highlight the potential of these strains to adhere to hydrophobic surfaces, a critical attribute for colonization and competitive exclusion of pathogens in probiotic applications.

### 3.7 Result of comparative analysis of hydrophobicity and auto-aggregation

**Fig 5:**
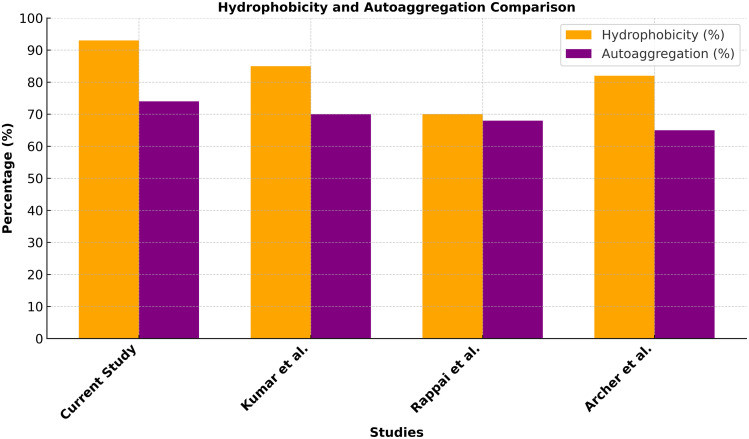
Comparative analysis of hydrophobicity and autoaggregation across various species. The graph displays the percentage of hydrophobicity (adhesion to hydrocarbons) and autoaggregation (self-aggregation). Italicized names represent the tested bacterial strains, including those from the current study and one representative high-performing strain from each reference study.

The comparative analysis of hydrophobicity and autoaggregation revealed substantial differences among the tested species (Fig 5). *Lb. paracasei IF5YR* and *Ent. faecium IF5RW2* exhibited the highest hydrophobicity (>90%) and autoaggregation (>80%), indicating strong adhesion to epithelial cells and robust biofilm formation potential. In contrast, *S. thermophilus* strains (*HN2* and *HN3*) displayed moderate hydrophobicity and lower autoaggregation, suggesting limited colonization potential. Notable differences were observed within the *Lactobacillus* genus, where *L. pentosus* and *L. delbrueckii* demonstrated lower hydrophobicity and autoaggregation compared to *L. paracasei*. *P. pentosaceus DM101* and *E. durans LAB18s* exhibited moderate adhesion but relatively lower autoaggregation compared to the current study’s strains (Fig 5). The selection of the best-performing individual isolate from each reference ensured a consistent and direct comparison of probiotic adhesion potential across species.

### 3.8 Bile salt hydrolase assay

A direct plate assay was utilized to evaluate bile salt hydrolase (BSH) activity in the probiotic cultures against two predominant conjugated bile salts: sodium deoxycholate and sodium taurocholate. The assay results, presented in Table 3, revealed clear zones surrounding the growth of both *Lb. paracasei* IF5YR and *Ent. faecium* IF5RW2, indicating active BSH enzyme production. The observed activity was comparable to that of the reference strains [11], demonstrating the ability of IF5YR and IF5RW2 to hydrolyse bile salts effectively.

**Table 3:** Results of bile salt hydrolase activity.

Isolates	Sodium deoxycholate	Sodium taurocholate
*Lb. paracasei* IF5YR	+	+
*Ent. faecium* IF5RW2	+	+

Note: Data presented here is qualitative and are based on the results of experiments performed in triplicates. “+” represents zone and “-” represents only growth without zone.

### 3.9 Antioxidant properties

**Fig 6:**
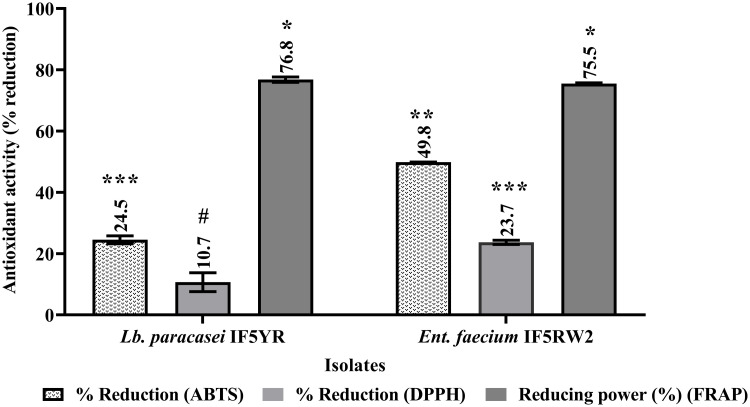
Antioxidant potential (FRAP, ABTS, DPPH) values of different isolates. Values are presented as Mean±SD. ^***#^P < 0.05: significantly different. Bars with common superscripts represent non-significance in the property.

This study evaluated the antioxidant properties of candidate probiotic isolates using three widely recognized assays: ABTS, DPPH, and FRAP. The results highlighted the strong reducing power of *Lb. paracasei* IF5YR and *Ent. faecium* IF5RW2, with FRAP values of 76.8 ± 0.8% and 75.5 ± 0.2%, respectively. As shown in Fig 6, the ABTS radical scavenging activity was comparatively lower, with *Ent. faecium* IF5RW2 demonstrating a higher reduction (49.8 ± 0.07%) than *Lb. paracasei* IF5YR (24.5 ± 1.3%). In the DPPH assay, the reduction percentages were 23.7 ± 0.7% and 10.7 ± 3.0% for *Ent. faecium* IF5RW2 and *Lb. paracasei* IF5YR, respectively, with significant differences between the two strains (p < 0.05).

Compared to the reference strain *Lb. rhamnosus* NDRI 184, the FRAP values of *Lb. paracasei* IF5YR and *Ent. faecium* IF5RW2 were comparable, demonstrating their similar reducing potential. These findings emphasize the antioxidant capabilities of these isolates, particularly their electron-donating capacity, and their potential utility in mitigating oxidative stress.

## 4 Discussion

The burgeoning global market for functional foods, particularly those enriched with probiotics, highlights the ongoing demand for probiotic strains with well-characterized functional properties. While traditional probiotic sources like dairy products have been extensively explored, emerging evidence highlights that human gut-derived probiotics may exhibit favourable functional traits, including enhanced resistance to gastrointestinal (GI) stresses and improved epithelial adhesion, making them exceptionally valuable for diverse dietary applications and individuals with specific sensitivities like lactose intolerance. Although the present study employed 16S rRNA sequencing for bacterial identification, advances in next-generation sequencing have revealed the extensive diversity of gut microorganisms, and future NGS-based investigations may further elucidate the genetic basis of functional traits relevant to health and pharmaceutical applications [9].

This study focused on the identification of potential probiotic candidates from infant fecal samples. From the isolated strains, *Lactobacillus paracasei* IF5YR and *Enterococcus faecium* IF5RW2 were selected as promising candidates based on phenotypic evaluation of probiotic-associated functional properties. This rigorous evaluation encompassed their capacity to survive harsh GI conditions, adhere to intestinal epithelia, and exhibit beneficial effects such as bile salt hydrolysis and potent antioxidant activity.

Safety is a fundamental requirement for probiotics intended for food applications, especially within the dairy sector. While antibiotic resistance is a concerning, widespread phenomenon in gut bacteria, including those from infants, our selected strains, *Lb. paracasei* IF5YR and *Ent. faecium* IF5RW2, demonstrated sensitivity or intermediate sensitivity to most clinically relevant antibiotics. This susceptibility profile suggests a favourable preliminary safety profile based on phenotypic antibiotic susceptibility testing. Furthermore, the absence of haemolytic activity in both isolates indicates a favourable in vitro safety profile. [24]. Given the dual role of *E. faecium* as both probiotic candidate and opportunistic pathogen, In the present study, safety evaluation was limited to phenotypic assays, and no genomic screening for virulence factors or antibiotic resistance genes was performed. Comprehensive genomic characterization, including whole-genome sequencing, is essential for regulatory approval and consumer safety and is recommended for future studies, particularly for *Enterococcus faecium*. This comprehensive safety evaluation supports the suitability of these strains for probiotic applications while recognizing the ongoing need for rigorous surveillance [7,9,10].

For probiotics to confer systemic benefits, surviving the acidic stomach (pH 1.5) and bile-rich intestine (2%) is essential. Both strains exhibited exceptional tolerance to these conditions, likely through mechanisms such as intracellular pH regulation and adapted metabolism. This resilience suggests the potential to withstand gastrointestinal stress under in vitro conditions; however, in vivo studies are required to confirm survival and colonization in the human gut. It should be noted that acid and bile tolerance were assessed under static *in vitro* conditions, which may not fully mimic the dynamic gastrointestinal environment; therefore, further validation using dynamic gastrointestinal models is warranted to confirm the survival potential of these strains.

Effective colonization also relies on adhesion influenced by autoaggregation and cell surface hydrophobicity. Our isolates demonstrated high autoaggregation (>70%) and notable hydrophobicity (>90% with xylene), indicating strong epithelial interaction potential. These adhesion-related properties indicate *in vitro* interaction potential, but their role in intestinal persistence or barrier function requires *in vivo* validation [25,26].

The observed bile salt hydrolase (BSH) activity in our selected strains is another significant functional attribute. BSH plays a crucial role in bile salt deconjugation, which can influence host cholesterol metabolism [19]. While some studies on *Lb. casei* strains have reported conflicting BSH activity; our findings demonstrate detectable BSH activity under the tested *in vitro* conditions in both *Lb. paracasei* IF5YR and *Ent. faecium* IF5RW2 [27]. This enzymatic capability suggests potential relevance in cholesterol metabolism; however, *in vitro* BSH activity alone does not guarantee cholesterol-lowering effects, and further *in vivo* studies are required to confirm any cardiovascular benefits.

Furthermore, *in vitro* antioxidant activity has been proposed as a desirable functional trait; however, its protective relevance in infants cannot be inferred without clinical evidence [28,29]. Our comprehensive antioxidant assessments revealed that *Lb. paracasei* IF5YR and *Ent. faecium* IF5RW2 possess robust antioxidant potential. The high FRAP values (>75%) underscore their strong reducing power, signifying an effective capacity to neutralize free radicals [30]. While ABTS and DPPH radical scavenging activities varied, consistent with strain-specific mechanisms, the overall antioxidant profile of our isolates compares favourably with previously reported probiotic strains [31,32]. This antioxidant capacity is likely mediated by the synthesis of bioactive compounds like exopolysaccharides and the activation of specific enzymatic pathways, suggesting a potential contribution to antioxidant activity through multiple mechanisms. *L. paracasei*’s established industrial role in fermented dairy products, coupled with the novel probiotic and antioxidant traits of IF5YR, strengthens its candidacy for functional food innovation [33]. These properties indicate potential applicability in functional food development; however, *in vivo* or clinical studies are required to confirm any specific health-related effects.

Finally, the integration of phylogenetic and comparative analyses aided in understanding the taxonomic placement and functional characteristics of our selected strains. Phylogenetic assessment based on 16S rRNA sequencing supported the taxonomic identification of the isolates at the species level and their phylogenetic relatedness to previously reported strains. This computational validation, coupled with experimental characterization, offers a holistic understanding of their probiotic potential. Furthermore, our study uniquely contributes to the understanding of probiotic diversity within the Indian context. Given the significant variations in gut microbiota composition across global populations due to distinct dietary habits and environmental factors, the identification and comprehensive characterization of novel, regionally relevant probiotic strains from Indian infants provide a robust foundation for developing targeted, effective functional dairy products tailored to the local population. The phenotypic characterization of gastrointestinal survival, adhesion, BSH activity, and antioxidant potential indicates promising probiotic-associated properties, warranting further genomic and *in vivo* validation.

## 5 Conclusion

This study demonstrates that *Lactobacillus paracasei* IF5YR and *Enterococcus faecium* IF5RW2, novel strains isolated from the infant gut, are exceptionally promising probiotic candidates. Their proven resilience to harsh gastrointestinal conditions, robust adhesion capabilities, confirmed bile salt hydrolase activity, and significant antioxidant potential collectively underscore their strong viability for functional food applications. Phylogenetic analysis based on 16S rRNA sequencing supported taxonomic identification, while phenotypic assays provided initial insight into probiotic-associated functional traits. This comprehensive experimental and computational framework not only provides a powerful template for future probiotic screening but also specifically highlights the immense potential of these strains for incorporation into the dairy industry, supporting their potential consideration for future functional dairy product development. Future efforts should focus on *in vivo* validation and targeted clinical trials to fully substantiate their health benefits, accelerating their translation into effective tools for gut health management and personalized nutrition.

## Supporting information

S1 Fig Comparative analysis. Numeric values of comparative analysis of acid, bile, cell autoaggregation and cell surface hydrophobicity in Figs 3 and 5.
(XLSX)

S2 Fig *In-vitro* CA and CSH of isolates. Numeric values of *in vitro* cell autoaggregation and cell surface hydrophobicity of isolates in Fig 4.
(XLSX)

S3 Fig Antioxidant potential of different isolates. Numeric values of Antioxidant potential (FRAP, ABTS, DPPH) values of different isolates in Fig 6.
(XLSX)
